# A clinical trial to evaluate the effect of the Mediterranean diet on smokers lung function

**DOI:** 10.1038/s41533-019-0153-7

**Published:** 2019-11-27

**Authors:** Francisco Martín-Luján, Roxana-Elena Catalin, Patricia Salamanca-González, Mar Sorlí-Aguilar, Antoni Santigosa-Ayala, Rosa Maria Valls-Zamora, Núria Martín-Vergara, Teresa Canela-Armengol, Victoria Arija-Val, Rosa Solà-Alberich

**Affiliations:** 1 Institut Català de la Salut, CAP El Morell. Institut Universitari d’Investigació en Atenció Primària-IDIAP Jordi Gol. School of Medicine and Health Sciences, Universitat Rovira i Virgili, Reus, Spain; 2 Institut Català de la Salut, CAP Bonavista, Carrer Set, 36, 43100 Tarragona, Spain; 3 Research Support Unit Tarragona-Reus, Institut Universitari d’Investigació en Atenció Primària-IDIAP Jordi Gol, Tarragona, Spain; 4 Institut Català de la Salut, CAP Sant Salvador, Institut Universitari d’Investigació en Atenció Primària-IDIAP Jordi Gol, School of Medicine and Health Sciences, Universitat Rovira i Virgili, Reus, Spain; 5 Hospital Universitari Sant Joan, Institut d’Investigació Sanitària Pere Virgili (IISPV), Functional Nutrition, Oxidation and Cardiovascular Disease (NFOC-SALUT) group, Universitat Rovira i Virgili, Reus, Spain; 6 Institut Català de la Salut, CAP Horts de Miró, Tarragona, Spain; 7 Institut Català de la Salut, CAP Dr Sarro, Tarragona, Spain; 8 Institut Universitari d’Investigació en Atenció Primària-IDIAP Jordi Gol, School of Medicine and Health Sciences, Universitat Rovira i Virgili, Reus, Spain

**Keywords:** Clinical trial design, Nutritional supplements

## Abstract

Data on the association between lung function and some dietary patterns have been published. However, it is not yet well known if whether the Mediterranean Diet (MD) pattern can preserve or improve lung function. Our purpose is to evaluate the effect of increased MD adherence on lung function in smokers. A multicenter, parallel, cluster-randomized, controlled clinical trial is proposed. A total of 566 active smokers (>10 packs-year), aged 25–75 years will be included, without previous respiratory disease and who sign an informed consent to participate. Twenty Primary Care Centres in Tarragona (Spain) will be randomly assigned to a control or an intervention group (1:1). All participants will receive advice to quit smoking, and the intervention group, a nutritional intervention (2 years) designed to increase MD adherence by: (1) annual visit to deliver personalized nutritional education, (2) annual telephone contact to reinforce the intervention, and (3) access to an online dietary blog. We will evaluate (annually for 2 years): pulmonary function by forced spirometry and MD adherence by a 14-item questionnaire and medical tests (oxidation, inflammation and consumption biomarkers). In a statistical analysis by intention-to-treat basis, with the individual smoker as unit of analysis, pulmonary function and MD adherence in both groups will be compared; logistic regression models will be applied to analyze their associations. We hope to observe an increased MD adherence that may prevent the deterioration of lung function in smokers without previous respiratory disease. This population may benefit from a dietary intervention, together with the recommendation of smoking cessation.

## Introduction

Nutrition and dietary habits are recognized factors in the development, progression and prevention of chronic pathologies such as cancer and cardiovascular diseases.^1,2^ However, the impact of diet on lung function and respiratory diseases is not as well established.^3^

Tobacco smoke is the most important factor in the etiopathogenesis of respiratory pathology, although other factors may also be involved, such as environmental agents, respiratory infections, genetic and epigenetic disorders, and dietary habits.^4^ Inhalation of tobacco smoke particles accelerates the physiological decline of lung volume attributable to aging and increases susceptibility to respiratory dysfunction.^5^ Smoking generates thousands of free radical particles, an important source of inflammation and oxidative stress, which can be neutralized by dietary intake of antioxidants or/an anti-inflammatory leading to a protective effect on lung function.^6^ Specifically, the protective action of certain nutrients and food on respiratory function parameters, such as forced vital capacity (FVC) and expiratory flow in the first second (FEV1), has been reported.^7^ Thus, consumption of fruits and vegetables with a high content of antioxidant vitamins, phenolic compounds, minerals and dietary fibre has a beneficial effect on respiratory health.^8,9^ Regarding the role of vitamins (vitamins C, D, E, A, beta and alpha carotene), they have been associated with improvement in features of chronic obstructive pulmonary disease (COPD) such as symptoms and exacerbations, and high intake would probably reduce the annual decline of pulmonary function.^10^ Omega-3 fatty acids present in fat fish and shellfish have an anti-inflammatory effect involved in the pathophysiology of COPD.^11^ In contrast, high consumption of processed meat has been associated with worse pulmonary function and increased risk of COPD.^12,13^ Also, low or moderate alcohol consumption has been associated with better lung function, while excessive intake has detrimental effects.^14,15^ Although the evaluation of the effect of individual foods has been valuable, it presents conceptual and methodological limitations because the diet contains a variety of foods that may interact with one another, thus varying the effects of single foods.^16^ Therefore, analysis of dietary patterns is now considered to offer a better approximation to the study of the effects of food on health. Available evidence shows that the “western-unhealthy” diet with high consumption of fried and processed foods, processed meats, refined sugars and sweets increases the risk of COPD,^17,18^ whereas a “prudent-healthy” diet rich in whole grains, vegetables, fruits and fish is associated with better lung function.^19^

The traditional dietary pattern of Mediterranean (MD, Mediterranian diet) countries includes a set of characteristic foods with beneficial effects against inflammation and oxidation, such as fat fish, vegetables, legumes and fresh fruits, nuts and olive oil.^20^ The MD is rich in antioxidants (vitamins C and E, beta-carotene and folates), phenolic compounds (the most abundant being flavonoids), and mono and polyunsaturated fatty acids.^21^ The effectiveness of the MD intervention for cardiovascular risk reduction in adults is well established, and for years has been recommended in cardiovascular prevention.^1^ Other studies have expanded the MD benefits to other diseases such as endocrine-metabolic, neurodegenerative and certain types of cancer.^22^ Extending this recommendation to respiratory pathology related to smoking is of evident interest in primary prevention.

The present study is part of a broader program to reduce smoking behaviours: the RESET-DIET project, a clinical trial on smoking cessation, designed to evaluate the efficacy of a motivational intervention based on information obtained from spirometry.^23^ In this project, our group carried out a pioneering observational study to identify dietary patterns present in an adult smoker population, and examine their association with lung function.^24^ We identified three major patterns: a mixed intake pattern with alcohol consumption (wine, beer and distillates), a westernized-style pattern (red and processed meat, dairy products and sugary drinks) and a Mediterranean-like pattern (poultry, eggs, fish, vegetables, legumes, potatoes, fruit and nuts). Multivariate analysis associated a higher probability of lung functional impairment with the mixed-alcohol pattern in both men and women (OR 4.56, 95% CI 1.58–13.18) and with the westernized-style pattern only in women (OR 5.62, 95% CI 1.17–27.02), compared to the Mediterranean-like pattern (OR 0.71, 95% CI 0.28–1.79).^24^ In contrast, a smaller sample power caused very wide intervals in the statistical tests (especially OR) and was not sufficient to achieve a significance inverse association (trend only) in this “prudent” pattern.

Altogether, the findings suggest that certain personal conditions (such as anthropometric characteristics) and dietary patterns in the smoking population are associated with impaired lung function, and offers new perspectives on avoiding the preventable loss of lung function through adherence to a healthy diet pattern.^24–27^ To our knowledge, no national or international groups are currently working on the protective effects of MD in preventing respiratory diseases. Therefore, our research purpose is to evaluate the effect of MD adherence on lung function in smokers without previous respiratory disease.

## Methods

### Study design

The MEDISTAR study (Mediterranean diet and smoking in Tarragona and Reus) is a multicenter, parallel, cluster-randomized, controlled clinical trial to be carried out in a sample of patients treated at primary health-care centres (Clinical Trial registry NCT03362372). This study is an extension of the DIET study, a pilot study carried out in 80 participants, with the aim to evaluate the effectiveness of an educational intervention to increase MD adherence, in a sample of smokers with no previous respiratory disease.^27^

**Fig. 1:**
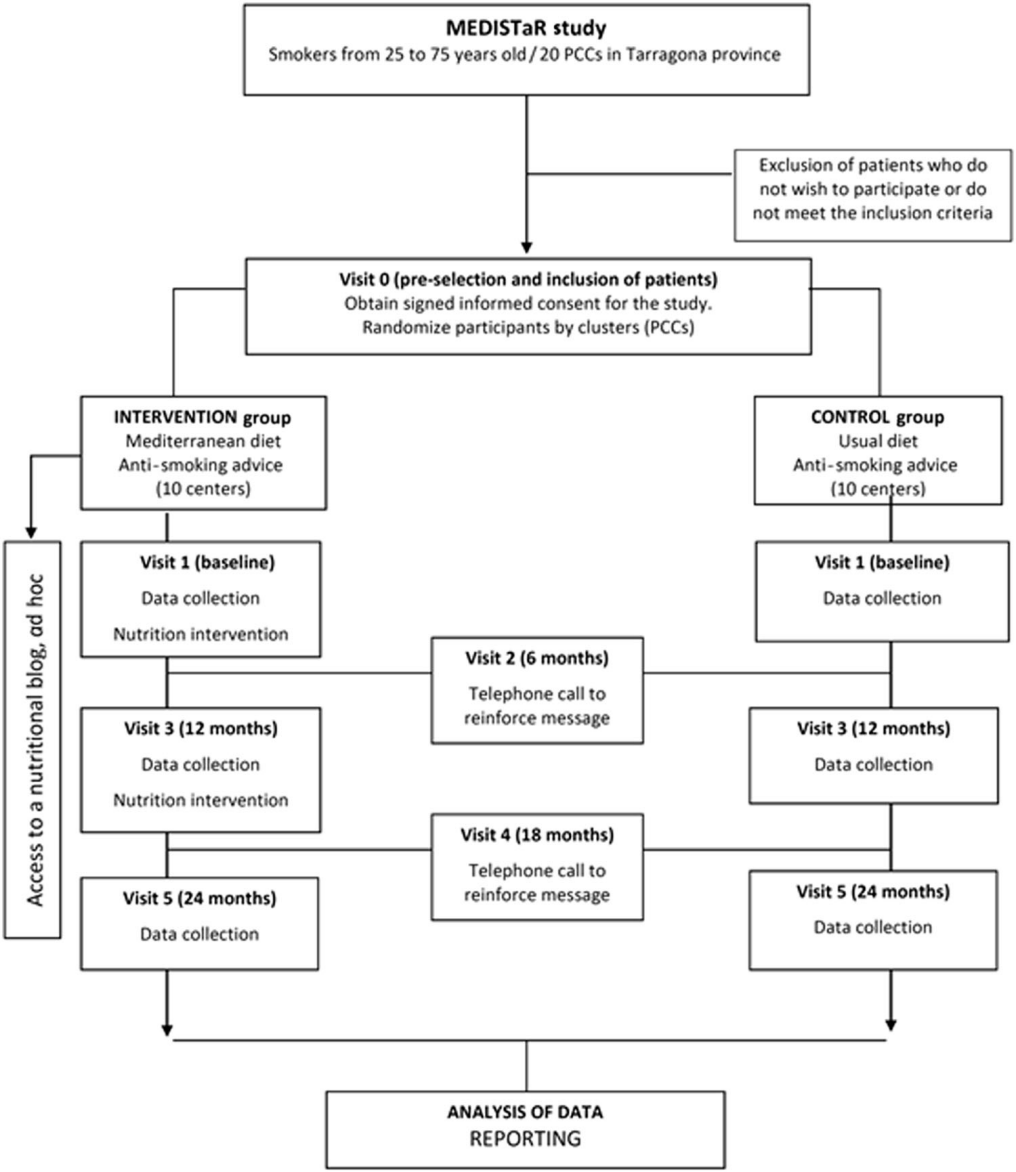
Project flowchart: participant selection, randomization and follow-up.

Figure 1 shows the flow diagram of the project (participant selection, randomization and follow-up) and Table 1 shows the diagram of visits with the activities carried out in each contact with the participants.

**Table 1:** Diagram of visits with the activities carried out in each contact with the participants.

	Study period						
	Enrollment	Allocation	Post-allocation				Close out
TImepoint	−T1	T0	T1	T2	T3	T4	
	−30 days	Day 0	6 months	12 months	18 months	24 months	
Activities	Randomization Selection inclusion	Collect data Intervention	Telephone tracking Support	Collect data Intervention	Telephone tracking Support	Collect data	Data analysis
Enrollment							
Eligibility screen	X						
Informed consent	X						
Allocation							
Data collection	X	X	X	X	X	X	
Basic physical exploration		X		X		X	
Complementary tests		X		X		X	
Diet questionnaires and diet		X		X		X	
Forced spirometry		X		X		X	
Consumption, inflammation/oxidation markers		X		X		X	
		X		X		X	
		X		X		X	
Intervention							
Control group: regular diet		X	X	X	X	X	
Intervention group: Nutrition Program in DM		X	X	X	X	X	

### Selection and randomization of participating centres

The study population will be recruited from patients treated at 20 primary health-care centres of Catalan Institute of Health (ICS, for its Spanish acronym) in Tarragona (Spain), which provide assistance to an adult population of ~280,000 inhabitants.

All participant centres will receive standardized formation about the methodology of study (4 h).

Participating centres will be assigned randomly to a control or intervention group (1:1), using a centralized process carried out by the Research Support Unit of the Institut d’Investigació en Atenció Primària-IDIAP Jordi Gol. This process will be made with a number list computer-generated for this purpose using EPIDAT 3.0 program.

### Criteria for patient recruitment

The inclusion criteria were age from 25 to 75 years old (both inclusive) and current smoker with cumulative consumption ≥10 packs-year. The exclusion criteria were a previous diagnosis of respiratory disease (such as COPD, asthma, bronchiectasis, pulmonary fibrosis, etc.), a medical history of chronic or terminal disorder that would affect the basal parameters (such as active cancer, severe heart or cerebrovascular disease, liver disease or kidney failure), or any problem or limitation that, at the discretion of the researcher, may be difficult to follow-up during the study period (such as mental illness).

During recruitment, all individuals will receive information about the study objectives and the activities related to their participation, and will sign an informed consent prior to their inclusion.

### Intervention

Candidates will be informed about the MEDISTAR study and offered the opportunity to participate. If they accept and meet the inclusion/exclusion criteria, they must sign an informed consent at the initial visit (V0).

Participants from the intervention centres will enter into an educational program to increase MD adherence. It will consist of five visits that integrate three operational components: (a) explain the details and benefits of following MD pattern in an individual visit of 25–30 min (V1). Data on the study variables will be collected and medical tests will be performed. This data gathering will be repeated annually for 2 years (V3 and V5); (b) Clarify any doubts about following the MD pattern and other advice received, in a telephone conversation. This annual contact will reinforce the intervention (V2 and V4); (c) Provide information about MD foods, cooking tips, adapted recipes, and other related topics in an ad hoc nutrition blog.

### Control group

The control group will carry out the same 5 visits (3 individual visits and 2 phone calls), although no changes will be made in their usual diet and they will not be provided with access to the nutritional blog.

All participants will receive health advice to stop smoking, according to the recommendation to health-care professionals in Catalonia: a proposal of smoking cessation expressed in a clear, firm and personalized way, in an empathic and respectful setting.

### Data collection

All the information will be collected in a computerized data collection questionnaire, only accessible from the administrators of the ICS corporate Intranet in Tarragona. Access will be restricted and controlled by a personal password for each researcher, who will be responsible for entering participant data.

The information contained in the questionnaire will be as follows:

- Socio-demographic data: sex, age, marital status, number of children, level of education and social class (according to the British General Register classification adapted for the Spanish population^28^).
- Medical history with treatment received (such as antihypertensives, statins, beta-blockers or inhaled drugs) or vitamin supplements, and respiratory symptomatology (such as dyspnoea, cough, expectoration, chest pain and other symptoms).
- Smoking habit: accumulated consumption (packs-year), current consumption (cigarettes/day), nicotine dependence (Fagerström test score^29^), motivation to quit smoking (Richmond test score^30^), and stage of change according to Prochaska and DiClemente model (precontemplation, contemplation, preparation, action, maintenance and relapse^31^).
- Alcohol consumption (frequency and grams of alcohol/week).
- Food consumption frequency and dietary intake information, collected using a 45-item food-frequency questionnaire (FFQ), validated for the Spanish population.^32^ The questions include the average frequency of food consumption using specific categories for number of times/week and times/month, during the previous 12 months.
- MD adherence, measured by administration of a previously validated 14-item questionnaire to assess compliance with dietary intervention in the PREDIMED study, a multicentre clinical trial aimed at assessing the MD effects on the primary prevention of cardiovascular disease.^33^ The questionnaire consists of 12 food consumption frequency questions and two about food intake habits considered characteristic of the MD. Each question scores 0 or 1, consequently, total score ranges from 0 to 14. The higher the score, the greater the degree of adherence to MD pattern. Therefore, a score of >9 is defined as “high” MD adherence, and a score of <7, as “low” MD adherence.
- Physical activity classification, measured with the short Catalan version of the International Physical Activity Questionnaire (IPAQ).^34^ According to the IPAQ guidelines, the participants were classified as engaged in vigorous physical activity, moderate physical activity and low physical activity.^35^
- Anthropometric values and adiposity markers: the height and body weight, measured when the participants wore indoor clothing without shoes. The waist circumference, measured at the mid-point between the lower border of the rib cage and the iliac crest, according to WHO-2011 recommendations. The body mass index (BMI), calculated according to the following formula: weight (kg)/height (m^2^). The waist circumference/height ratio. The conicity index, calculated according to the following equation: waist circumference/(0.109 × square root of (weight/height)).
- Physical examination: The blood pressure, measured twice in sitting position on the right arm, calculated as the mean value of the two measurements (using Omron M6 (Omron Healthcare Europe, Hoofddorp, The Netherlands)). The 12-lead electrocardiographic record (using PageWriter TC20 (Philips Medical Systems, Andover, USA)).
- Exhaled carbon monoxide concentration, determined by CO-oximetry (using MicroCO™ (Medical Device Depot, Ellicott City, USA)), that detects levels range of 0–100 ppm, with a sensitivity of 1 ppm.
- Pulmonary function: The FVC, the FEV1, and the relationship between them (FEV1/FVC), measured by spirometry (using spirometer model DATOSPIR-600© (SIBELMED, Barcelona, Spain) with a disposable Lilly-type transducer). The standard procedure is performed in agreement with the ATS/ERS recommendations:^36^ At least three tests will be performed, from which the best values for FVC and FEV1 should be selected. The test duration should be >6 s and the variability < 5%. Secondly, 400 µg of salbutamol are administered, and test is repeated after 20–30 min to evaluate the bronchodilator airway response. All tests will be sent to a single observer, to evaluate quality control and interpret the results through a computer program.
- Levels of glucose, total cholesterol and fractions, triglycerides, creatinine and transaminases were measured by standardized methods on an autoanalyzer (Beckman Coulter-Synchron, Galway, Ireland) in serum samples.
- Inflammation biomarkers were determined in serum samples by standardized automated methods. Briefly, high sensitivity C-reactive protein (hsCRP) was measured by automated immunoturbidimetry (Roche Diagnostics Systems, Madrid, Spain), and interleukin (IL) 6 was determined by Elisa kits (Abcam, Cambridge, UK).^37,38^
- Oxidation markers: oxidized LDL in EDTA plasma was measured with an ELISA kit (Mercodia AB, Uppsala, Sweden).^39^
- Food consumption markers: total polyphenol excretion (TPE) was determined in urine samples by means of the Folin-Ciocalteau method using an Oasis® MAX 96-well plate cartridge for solid-phase extraction.^40^

### Sample size and power calculation

Initially, the sample size has been calculated for a simple randomized design, using the ARCSINUS approximation. Thus, in a bilateral contrast, accepting a risk *α* = 0.05, a risk *β* < 0.2 and a follow-up loss rate of 25%, 192 subjects are required in each group to detect significant differences between two proportions, estimating that this would be 0.17 in group-1 and 0.31 in group-2.^24^ Accepting that the effect of the design is 1.7, applying an intraconglomerate correlation coefficient <0.05, a sample of 566 participants will be needed, 283 in each group.^41^

### Study variables

The main variable of interest is impaired lung function. This alteration will be defined according to the guidelines of the American Thoracic Society and The European Respiratory Society:^41^ an anomalous FVC% or FEV1% was defined as a value lower than 80% of the predicted. In addition, a FEV1/FVC ratio <70% will be considered altered. In this way, we get three possible spirometric patterns:

- A non-obstructive (restrictive) alteration: a FVC% > 80% of the predicted value and a FEV1/FVC ratio > 70%
- An obstructive alteration: a FVC% > 80% of the predicted value and a FEV1/FVC ratio < 70%
- A mixed alteration: a FVC% > 80% of the predicted value and a FEV1/FVC ratio < 70%

In addition, according to GOLD (the Global Initiative for Chronic Obstructive Pulmonary Disease), a FEV1/FVC ratio < 70% in a post-bronchodilator test also is defined as COPD.^42^ Significant changes in the FVC and FEV1 values between the initial and final visit (>12% and 200 mL) will be also considered, as decreases beyond what is expected in smokers.

As secondary variables, anthropometric and adiposity markers (weight, height, BMI, waist circumference, waist circumference/height ratio and conicity index), dietary patterns (adherence and consumption data) and serum inflammation and anti-oxidation biomarkers will be evaluated. All variables will be determined at baseline and at the end of follow-up, in control and intervention groups.

### Statistical analysis

Data will be extracted from the centralized database for blinded analysis, based on intention to treat. The efficacy of randomization will be assessed by comparing the homogeneity of the intervention and control groups. Losses to follow-up will be evaluated to determine if they have occurred independently of the allocation group.

Categorical variables will be described as frequencies or percentages, and continuous variables by the mean and standard deviation or inter-quartile range. Analysis will be stratified by study groups and compared at basal level using the *χ*^2^ test, the Student's *t* or Mann–Whitney *U* test.

Pulmonary function, anthropometric data, foods consumed and MD adherence will be compared at initial (V1) and final (V5) visits. A multivariate analysis will be performed to determine the factors independently associated with the observed changes, adjusting for relevant covariates (such as sociodemographic and anthropometric parameters, physical activity, alcohol consumption or use of drugs that can modify lung function). The results will be presented as hazard ratios, with 95% confidence intervals. All analysis will be performed with the SPSS v22.0 program, considering a *p*-value < 0.05 statistically significant.

### Ethical considerations

The MEDISTAR study will follow the principles set out in the Declaration of Helsinki and the ethical and scientific quality standard of the International Conference on Harmonization guidance, E6(R2) for Good Clinical Practice. It also fulfils the requirements established in the legislative framework in Spain for the field of biomedical research, the protection of personal data and bioethics.

The ethical and scientific quality of the study has already been evaluated by the Ethics and Scientific Committees of IDIAP-Jordi Gol under the code P17/089, incorporating its recommendations and suggestions.

## Discussion

The present study proposes a nutritional intervention, an unconventional strategy in the respiratory pathology approach, with a pragmatic design that take into account the conditions of clinical practice in primary health care.^43^ However, the intervention may be less intensive than that described in cardiovascular prevention studies.^44^ It focuses on reinforcing the consumption of foods considered as protective in the typical diet of the Mediterranean region and reducing the consumption of foods in the standard Western diet that have harmful effects on pulmonary physiology. If the efficacy of this intervention can be demonstrated, it could be readily implemented in clinical practice.

The abilities and attitudes in dietetics and nutrition of health-care professionals can be a difficulty to consider when planning to implement a nutritional intervention.^45,46^ Knowledge and skills are essential to properly evaluate dietary intake with specific questionnaires, as well as to increase the validity and reliability of nutritional education. Therefore, in order to standardize the intervention, the dieticians who collaborate in the MEDISTAR study and who design the intervention and develop the educational content and materials will provide thorough training in MD and pulmonary function at the beginning of the study for all participating health-care professionals.

The effect on lung function will be assessed by comparing the results of several forced spirometry tests, taken during the project follow-up period, between intervention and control groups. Although this should be sufficient time to observe some effect of the diet on spirometry values, it may be too short to show statistically and clinically significant differences between the two study groups. In any case, the results should provide additional evidence of the MD effect on lung function in a population without respiratory disease.

A common limitation of long-term projects is the loss of participants during the follow-up. To minimize losses, a multi-component intervention incorporating communication technology has been planned. In this sense, the use of a nutritional blog is intended to facilitate interaction between the intervention group participants and the researchers, beyond the scope of the office visit, and should favour adherence during the study period. Moreover, annual telephone contacts should help to consolidate the intervention and maintain direct personal contact with all participants in both study groups.

The MEDISTAR study also has important strengths. It takes a multidisciplinary approach to the serious health problem caused by tobacco use, integrating elements of epidemiological, clinical and basic research. In addition, the research team includes professionals with complementary experience in conducting clinical trials of lifestyle interventions. The intervention is carefully structured to determine its effect on the main clinical outcomes and maximize its transferability to the primary health-care setting. This highly innovative project offers a new paradigm of nutritional recommendations to address pulmonary dysfunction in smokers. This approach, drawing on a field of nutrition that has scarcely been studied and online new technology that is already familiar to much of the study population, may contribute to better adherence to the intervention.

The scientific community pays increasing attention to the identification of modifiable risk factor for prevention and treatment of the respiratory disease.^42^ According to the available evidence, some foods and nutrients, especially those with antioxidants and anti-inflammatory properties, have been associated with improved lung function and lower risk of COPD, when they are part of a prudent-healthy dietary pattern.^47^ But several studies in the general population and in patients with respiratory pathology have reported that the current dietary intake are becoming unhealthy, so nutritional intervention could be a good opportunity to implement early primary prevention strategies.

There is no exclusive prudent diet identified as healthiest for respiratory health. The MD is a diet rich in food with healthy fats and natural antioxidants, and could be a role model for its positive effects on lung function. This is the final purpose of the MEDISTAR intervention study: to provide health professionals with new evidence on the MD effects on the respiratory health protection in subjects without respiratory disease. If the results confirm our hypothesis that an increased MD adherence may prevent the deterioration of lung function in smokers, it could have a great potential for preventive recommendation in general population, as part of a healthier lifestyle, especially in smokers who cannot quit smoking.

### Reporting Summary

Further information on research design is available in the Nature Research Reporting Summary linked to this article.

## Supplementary information

Reporting summary

MEDISTAR PROTOCOL

CONSORT Check-list

## Data Availability

Full datasets are not publicly available at this point to protect anonymity of participants to ensure data security until the MEDISTAR project is completed. After project completion, selected data can be made available.
