# Supplementary material for: A clinical trial to evaluate the effect of the Mediterranean diet on smokers lung function
Source: NPJ Prim Care Respir Med. 2019 Nov 27;29:40. doi: 10.1038/s41533-019-0153-7 (PMC6881294; doi:10.1038/s41533-019-0153-7)
Supplement: Supplementary file 2 — MEDISTAR PROTOCOL [file 41533_2019_153_MOESM2_ESM.docx]

**RESEARCH PROTOCOL**

**MEDISTAR STUDY**

**(Mediterranean Diet and Smoking in Tarragona and Reus)**

**Effect of the Mediterranean diet on lung function in smokers without previous respiratory disease**

**
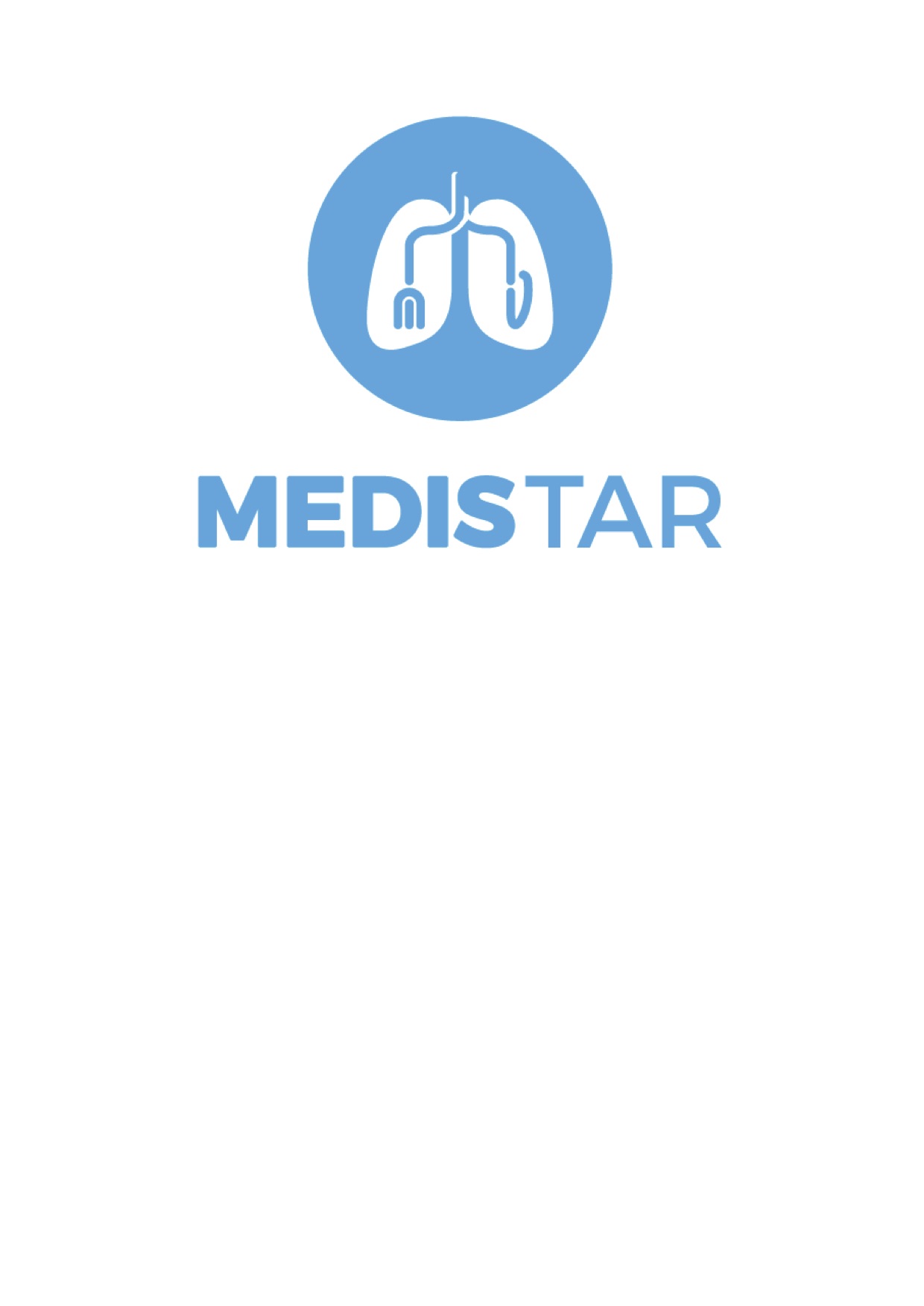
**
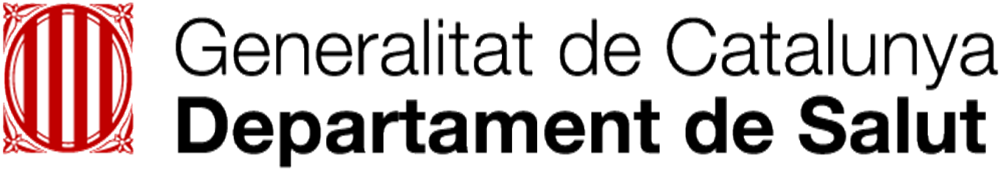

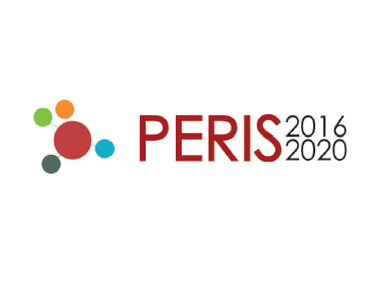


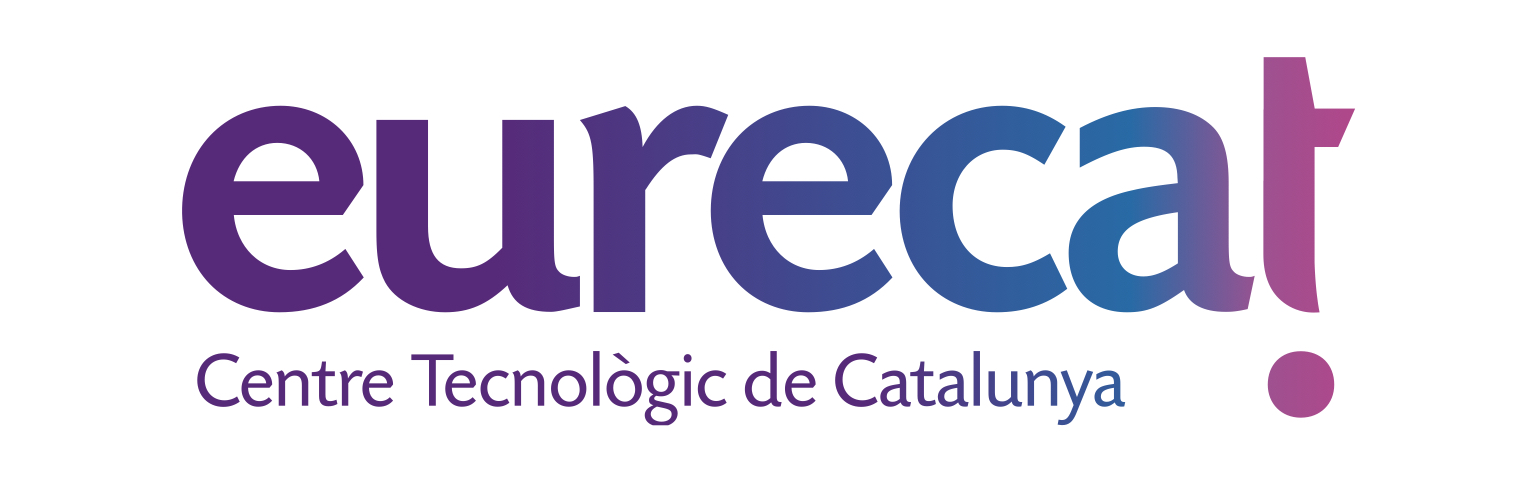

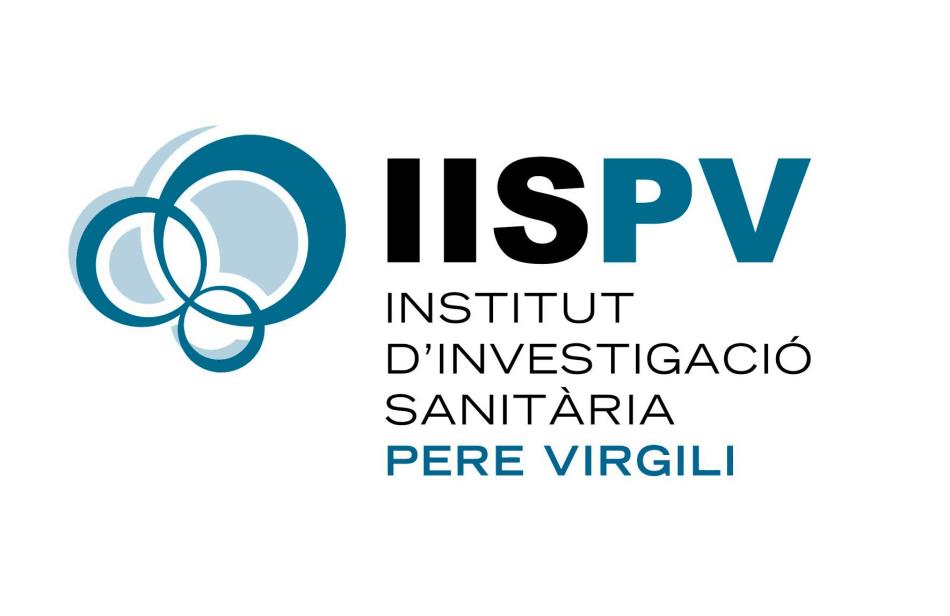

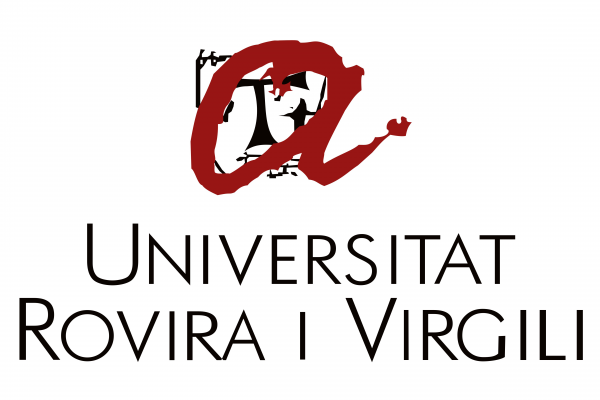

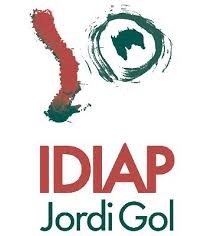

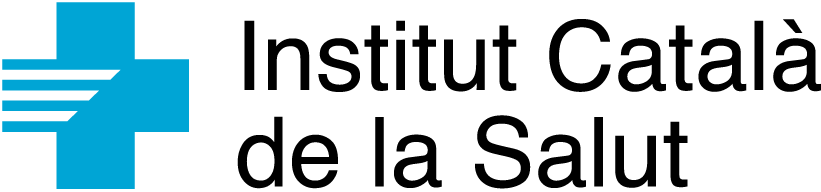


**RESEARCH TEAM**

**Principal Investigator**

Francisco Martín Luján

**Co-investigators**

Roxana-Elena Catalin

Patricia Salamanca González

Mar Sorli Aguilar

Antoni Santigosa Ayala

Rosa Maria Valls Zamora

Mª Victoria Arija Val

Mª Teresa Canela Armengol

Rosa Solà Alberich

**Research support team (IDIAP-JG Reus-Tarragona)**

Josep Basora Gallisà

Cristina Rey Reñones

**CONTENTS**

1. Summary 4

2. Abbreviations 5

3. Project description 6

3.1. Background of the topic

3.2. Preliminary results

3.3. Hypothesis and objectives

3.3.1. Hypothesis

3.3.2. Primary objective

3.3.3. Secondary objectives

4. Methodology and work plan 10

4.1. Design

4.2. Participants

4.3. Calculation of the sample

4.4. Interventions

4.5. Data collection

4.6. Study variables

4.7. Statistical analysis

5. Timeline 14

6. Limitations and strengths of the study 16

7. Ethical aspects 18

8. Available instrumentation and facilities 19

9. Funding sources 19

10. References 20

11. Annexes and figures 22

1. **SUMMARY**

Recent studies have shown the impact of eating patterns on lung function. However, the specific benefits of the Mediterranean Diet (MD) in preventing pulmonary dysfunction are not well known.

**Objective.** To evaluate the effect of MD adherence on the deterioration of lung function in smokers.

**Methodology.** Design: Controlled, parallel, multicenter, cluster-randomized clinical trial. Participants: 566 active smokers aged 25 to 75 years with a cumulative consumption of more than 10 packets/year, without previous respiratory disease and who agree to the conditions of the study and sign an informed consent to participate. Scope: 20 Primary Care Centers managed by the Catalan Health Institutes in Tarragona Province, to be randomly assigned to a control or an intervention group (1:1). All participants will receive advice to quit smoking. Intervention: A 2-year nutritional intervention designed to increase MD adherence. Elements include: 1) annual visit to deliver personalized nutritional education, 2) annual telephone contact to reinforce the intervention, and 3) access to an ad hoc online dietary blog. Control group: Continuation of usual diet pattern. Assessment (both groups, initially and annually for two years): a) pulmonary function, evaluated by forced spirometry and b) adherence to the MD, evaluated by a 14-item questionnaire and medical tests. Statistical analysis: Intention-to-treat basis, with the individual smoker as unit of analysis. Pulmonary function and adherence to a Mediterranean-style diet in both groups will be compared.

**Expected results.** MD adherence prevents the deterioration of lung function in smokers without previous respiratory disease. Thus, MD is key to pulmonary disease prevention, together with the recommendation of smoking cessation.

1. **ABBREVIATIONS**

PCC: Primary care center

MD: Mediterranean diet

FEV1: Expiratory flow in the first second

FVC: Forced vital capacity

ICS (Catalan acronym): Catalan Institute of Health

IDIAP - Jordi Gol (Catalan acronym): Research Institute for Primary Care - Jordi Gol

BMI: Body mass index

COPD: Chronic obstructive pulmonary disease

MEDISTAR: MEditerranean DIet and Smoking in Tarragona and Reus

AbC: Abdominal circumference

PERIS (Catalan acronym): Strategic Plan for Research and Innovation in Health

DCQ: Data collection questionnaire

RESET: REsults of Spirometry to Encourage Tobacco cessation

**3. PROJECT DESCRIPTION**

**3.1. BACKGROUND OF THE TOPIC**

Nutrition and dietary habits are recognized factors in the development, progression, and prevention of chronic pathologies such as cancer and cardiovascular diseases [1,2]. However, the impact of diet on lung function and respiratory diseases is not as well established [3].

In our environment, smoking is the main factor in the etiopathogenesis of respiratory pathology, although other factors may also be involved, such as environmental agents, respiratory infections, genetic disorders, and dietary habits [4]. Inhalation of tobacco smoke particles accelerates the physiological decline of lung volume attributable to aging and increases susceptibility to respiratory dysfunction [5]. For years, it has been known that tobacco smoke generates thousands of free radical particles, an important source of oxidative stress and inflammation.

This effect can be neutralized by the action of enzymatic antioxidants such as superoxide dismutase, catalase, or glutathione peroxidase. Dietary intake of other antioxidants also may have a protective effect on lung function [6]. Specifically, evidence of the protective action of certain food and nutrients on pulmonary ventilation parameters such as forced vital capacity (FVC) and expiratory flow in the first second (FEV1) has been reported [7]. Thus, consumption of fruits and vegetables with a high content of antioxidant vitamins, phenolic compounds, minerals, and dietary fiber has a positive effect on respiratory health [8-9]. Omega-3 fatty acids present in blue fish and shellfish have beneficial effects on the inflammatory mechanisms involved in the pathophysiology of chronic obstructive pulmonary disease (COPD) [10]. In contrast, high consumption of processed meat has been associated with worse pulmonary function and increased risk of COPD [11-12]. Also, low or moderate alcohol consumption has been associated with better lung function, while excessive intake has detrimental effects [13-14].

Although the evaluation of the effect of individual foods has been valuable, it presents conceptual and methodological limitations because the diet contains a variety of foods that may interact, with varying effects [15]. Therefore, analysis of dietary patterns is now considered to offer a better approximation to the study of the effects of food on health. Available evidence shows that the "western" diet with high consumption of fried and processed foods, processed meats, refined sugars, and sweets increases the risk of COPD [16-17] and that a "healthy" diet rich in whole grains, vegetables, fruits, and fish is associated with better lung function [18]. In this context, our group carried out a pioneering observational study that found a Mediterranean-type pattern is more likely to preserve lung function in smokers without previous respiratory disease, compared to a western diet, and a pattern of alcohol use was associated with an increased probability of functional impairment [19-21]. Therefore, adherence to a healthy dietary pattern, in addition to smoking cessation, offers new perspectives on avoiding the preventable loss of lung function in smokers.

The traditional dietary pattern of Mediterranean countries includes a set of characteristic foods with beneficial effects on inflammation and oxidation, such as legumes, fresh fruits and vegetables, blue fish, olive oil and nuts. The MD is rich in antioxidants (vitamin C and E, beta-carotene and folates), phenolic compounds (the most abundant being flavonoids), and monosaturated and polyunsaturated fatty acids [22]. This pattern has been associated with the reduction of cardiovascular events and all-cause mortality, and for years has been recommended in cardiovascular prevention. Extending this recommendation to respiratory pathology related to smoking is of evident interest in primary prevention.

To our knowledge, no national or international groups are currently working on the protective effects of MD in preventing respiratory diseases. Therefore, a clinical trial is presented with the objective of evaluating the impact on lung function of an intervention to increase MD adherence in a cohort of smokers without previous respiratory pathology.

**3.2. PRELIMINARY RESULTS**

The present study is part of a broader program to reduce smoking behaviors developed by the Study Group on Respiratory Pathology in Tarragona, accredited by IDIAP Jordi Gol (the Catalan acronym for the Jordi Gol Institute for Primary Care Research). The RESET study is a clinical trial on smoking cessation, designed to evaluate the efficacy of a motivational intervention based on information obtained from spirometry (Project FIS PI 11/01962) [19]. Within the RESET study, a two-phase project was designed. In the first phase, the RESET-DIET observational study, researchers identified the main dietary patterns present in the smoking population attended at primary care centers (PCCs) in Catalonia, and examined their association with lung function. Their results are already in an advanced stage of publication [20-21].

The RESET-DIET study identified three main dietary patterns: alcohol consumption (wine, beer and distillates), a westernized pattern (red and processed meat, dairy products and sugary drinks) and a Mediterranean pattern (poultry, eggs, fish, vegetables, legumes, potatoes, fruit and nuts). Multivariate analysis associated changes in respiratory function with the alcohol consumption pattern in both men and women (OR 4.56, 95% CI 1.58-13.18) and with the westernized pattern only in women (OR 5.62, 95% CI 1.17-27.02). In contrast, the Mediterranean pattern trended toward an inverse association with functional deterioration, without reaching statistical significance (OR 0.71, 95% CI 0.28-1.79) [20]. It was also observed that the inverse relationship with lung function is influenced by personal characteristics, such as abdominal obesity, especially in men [21].

These findings suggest that certain personal characteristics and dietary patterns in the smoking population are associated with impaired lung function. In addition to recommending smoking cessation, health professionals should consider an intervention regarding dietary factors. This is precisely the basis for the second-phase project, the MEDISTAR study (Mediterranean Diet and Smoking in Tarragona and Reus), detailed in the following section.

**3.3. HYPOTHESIS AND OBJECTIVES**

**3.3.1. Hypothesis**

Increased adherence to the MD pattern in addition to smoking cessation advice, will prevent the impairment of lung function in smokers without respiratory disease.

**3.3.2. Primary objective**

To evaluate the effect of MD adherence on the deterioration of lung function in smokers without previous respiratory disease treated in Tarragona PCCs.

**3.3.3 Secondary objectives**

In smokers without previous respiratory disease:

1) To evaluate the effectiveness of a multicomponent nutritional educational intervention designed to increase adherence to the MD pattern.

2) To study the relationship between lung function and anthropometric parameters such as the abdominal circumference (AbC) and body mass index (BMI).

3) To study the relationship between lung function and various markers of food consumption, inflammation and oxidation.

**4. METHODOLOGY AND WORK PLAN**

**4.1. DESIGN**

A multicenter, cluster-randomized, controlled clinical trial aimed at assessing the effect on lung function of increasing MD adherence in a cohort of smokers without previous respiratory pathology. (Clinical Trial registry with Nº :NCT03362372).

Figure 1 shows the flow diagram of the project.

**4.2. PARTICIPANTS**

The study population will be recruited from patients treated at 20 PCCs managed by the Catalan Institute of Health (ICS) in Tarragona, which provide assistance to an adult population of approximately 280,000 inhabitants.

Participants who give written informed consent to participate and meet the following criteria will be included:

- Active smoker with cumulative consumption ≥10 pack-years

- Age between 25 and 75 years, inclusive

- Internet access

Participants with any of the following criteria will be excluded:

- History of respiratory disease

- Chronic or terminal disorder that affects basal parameters

- Any limitation to follow-up participation

**4.3. CALCULATION OF THE SAMPLE**

The sample size was initially calculated for a simple randomized design, using the ARCSINUS approximation. Thus, in a bilateral contrast, accepting a risk α = 0.05, a risk β <0.2 and a follow-up loss rate of 25%, 192 subjects are required in each group to detect significant differences between two proportions, estimating that this would be 0.17 in group-1 and 0.31 in group-2 [20]. Accepting that the effect of the design is 1.7, applying an intraconglomerate correlation coefficient <0.05 [23], a sample of 566 participants will be needed, 283 in each group (38 volunteers from each participating PCC).

**4.4. INTERVENTIONS**

Participating centers will be assigned randomly to a control or intervention group (1:1). Volunteers will be referred to an initial visit (V0) to confirm inclusion criteria and sign an informed consent (Annex 1). Basal variables will be collected at that time.

Participants from the intervention PCCs will enter into an educational program to increase adherence to the MD. It will consist of five contacts that integrate three operational components:

a) Explain the details of the MD and the benefits of following this dietary pattern in an individual visit of 25-30 minutes (V1). Data on the study variables will be collected and medical tests will be performed. This data gathering will be repeated annually for two years (V3 and V5).

b) Clarify any doubts about following the MD pattern and other advice received, in a telephone conversation. This annual contact will reinforce the intervention (V2 and V4).

c) Provide information about MD foods, cooking tips, adapted recipes, and other related topics in an ad hoc nutrition blog.

The control group will carry out the same 5 visits (3 individual visits and 2 phone calls), although no changes will be made in their usual diet and they will not be provided with access to the nutritional blog.

All participants will receive health advice to stop smoking, according to the protocol recommended to health professionals in Catalonia: a proposal of smoking cessation expressed in a clear, firm, and personalized way, in an empathic and respectful setting.

At the end of the study, if the intervention is effective, control group members will be invited to participate in the same intervention program.

**4.5. DATA COLLECTION**

All the information will be collected in a computerized data collection questionnaire (DCQ), only accessible from the administrators of the ICS corporate Intranet in Tarragona. Access will be restricted and controlled by a personal password for each researcher, who will be responsible for entering participant data.

This DCQ will include initial information (V0):

- Socio-demographic data (age, sex, marital status, number of children, level of education, social class)
- Medical history and respiratory symptomatology (dyspnea, cough, expectoration, chest pain, other symptoms)

At follow-up visits (V1, V2 and V3), the following information will be obtained:

- Physical examination (weight, height, BMI, blood pressure, and level of exhaled carbon monoxide determined by CO-oximetry)
- Smoking habit (accumulated consumption, current consumption, nicotine dependence, motivation to quit smoking)
- Alcohol consumption
- Physical Activity Classification Questionnaire [24]
- Food frequency questionnaire to assess food consumption [25]
- MD adherence, based on 14-item questionnaire [26]
- 12-lead electrocardiogram
- Forced spirometry and bronchodilator test to measure FVC, FEV and the ratio between them (FEV1/FVC).
- Levels of glucose, total cholesterol and fractions, triglycerides, creatinine and transaminases (serum, autoanalyzer)
- Serum inflammation markers: high-sensitivity C-reactive protein and interleukin-6 (serum, autoanalyzer and assay ELISA)
- Oxidation markers: oxidized LDL in plasma (plasma EDTA, assay ELISA)
- Food consumption markers: excreted of total polyphenols (fresh urine, Folin-Ciocaletau)

**4.6. STUDY VARIABLES**

A) The main variable of interest is impaired lung function at the end of follow-up, defined as at least one of the following values: FVC <80%, FEV1 <80%, and FEV1/FVC <0.7 [27]. As secondary variables, spirometry tests (FVC and FEV1), anthropometrics (BMI and AbC), and dietary patterns (adherence and diameter consumption markers) will be evaluated at the end of follow-up.

**4.7. STATISTICAL ANALYSIS**

Data will be extracted from the centralized database for blinded analysis, based on intention to treat. The efficacy of randomization will be assessed by comparability and homogeneity of the intervention and control groups. Losses to follow-up will be calculated for each group and evaluated to determine whether the proportion of losses is independent of assignment to the intervention or control group.

Qualitative variables will be described as frequencies or percentages, and quantitative variables by the mean and standard deviation or interquartile range. Analysis will be stratified by study groups and compared at basal level using the χ2 test for qualitative and the Student t or Mann-Whitney U test for quantitative variables.

Pulmonary function, anthropometric data, foods consumed, and MD adherence at V1 will be compared to values obtained at the final visit (V5). A multivariate analysis will be performed to determine the factors independently associated with the observed changes, adjusting for relevant covariates. The results will be presented as hazard ratios, with 95% confidence intervals. All analysis will be performed with the SPSS v22.0 program, considering a p-value <0.05 statistically significant.

**5. TIME LINE**

Phases of project development, with activities and tasks to be developed by the entire research team:

**Preparation phase**

**Activity 1-** Registration and publication of study protocol

**Activity 2-** Design of questionnaires and nutrition education material

**Activity 3-** Design and development of the nutritional blog

**Activity 4-** Presentation of the project to PCCs

**Activity 5-** Training of Associate Researchers (PCC health professionals)

**Implementation and follow-up phase**

**Activity 7-** Recruitment of patients. Beginning of the intervention

**Activity 8-** Data collection, introduction to DCQ

**Activity 9-** Conduct follow-up visits (in person and by telephone)

**Data analysis and final evaluation**

**Activity 10-** Periodic quality analysis of records

**Activity 11-** Statistical analysis. Communication and publication of intermediate results

**Activity 12-** Statistical analysis. Communication and publication of final results

The schedule of tasks and data collection at each visit during the implementation and follow-up phase is detailed below.

|  |  |  |  |  |  |  |
| --- | --- | --- | --- | --- | --- | --- |
|  | **Visit 0** | **Visit 1** | **Visit 2** | **Visit 3** | **Visit 4** | **Visit 5** |
| **Study timeline** | - 30 days | Day 0 | 6 months | 12 months | 18 months | 24 months |
| **Aims** | Randomization  Selection  Inclusion | Data collection  Intervention | Phone  follow-up  Reinforcement | Data collection Intervention | Phone  follow-up  Reinforcement | Data collection |
| **Procedures** |  |  |  |  |  |  |
| Informed consent | **X** |  |  |  |  |  |
| Online data collection (DCQ) | **X** | **X** | **X** | **X** | **X** | **X** |
| Basic physical examination |  | **X** |  | **X** |  | **X** |
| Laboratory tests |  | **X** |  |  |  |  |
| Food questionnaires and dietary information |  | **X** |  | **X** |  | **X** |
| Forced spirometry |  | **X** |  | **X** |  | **X** |
| Inflammation / oxidation markers |  | **X** |  | **X** |  | **X** |
| **Intervention** |  |  |  |  |  |  |
| Control group: usual diet, usual clinical consultations, telephone follow-up |  | **X** | **X** | **X** | **X** |  |
| Intervention Group: MD Nutrition Program |  | **X** | **X** | **X** | **X** |  |
|  |  |  |  |  |  |  |

**6. LIMITATIONS AND STRENGTHS OF THE STUDY**

The present study has several limitations. The most important is related to the study population, because inclusion criteria may limit successful recruitment.

The intervention may be less intensive than that described in cardiovascular prevention studies [28]. However, it is an innovative strategy in the prevention of respiratory pathology and its pragmatic design take into account the actual working conditions in daily clinical practice in primary health care. It focuses on reinforcing the consumption of foods considered as protective in the typical diet of the Mediterranean region and reducing the consumption of foods in the standard Western diet that have harmful effects on pulmonary physiology. If the efficacy of this intervention can be demonstrated, it could be readily implemented in clinical practice.

Another limitation to take into account is the knowledge about dietetics and nutrition and the skills of the collaborating health professionals. Knowledge and skills are essential to properly evaluate MD adherence in terms of specific foods, as well as to increase the validity and reliability of the information collected using the study questionnaires and nutritional education. Therefore, in order to standardize the intervention, the dieticians who collaborate in the MEDISTAR study and who design the intervention and develop the educational content and materials will provide thorough training in MD and pulmonary function at the beginning of the study for all participating health professionals.

The effect on lung function will be evaluated by comparing the results of three consecutive forced spirometry measurements, taken annually during the three-year project period. Although this should be sufficient time to allow observations about the longitudinal effect of the diet on spirometry values, it could be too short to show statistically and clinically significant differences between the two study groups. In any case, these results should provide additional evidence of the effect of a MD on lung function in a population of smokers with no previous respiratory disease.

A common limitation of long-term projects is the loss of participants during the follow-up. To minimize losses, a multicomponent intervention involving technology has been designed. The use of a nutritional blog is intended to facilitate interaction between participants in the intervention group and the researchers, beyond the scope of the office visit, and should favor adherence during the study period. Moreover, annual telephone contacts should help to consolidate the intervention and maintain direct personal contact with all participants in both study groups.

The MEDISTAR study also has important strengths. It takes a multidisciplinary approach to the serious health problem caused by tobacco use, integrating elements of epidemiological, clinical, and basic research. In addition, the research team includes professionals with complementary experience in conducting clinical trials of lifestyle interventions. The intervention is carefully structured to determine its effect on the main clinical outcomes and maximize its transferability to the primary health care setting.

This highly innovative project offers a new paradigm of nutritional recommendations to address pulmonary dysfunction in smokers. This approach, drawing on a field of nutrition that has scarcely been studied and online new technology that is already familiar to much of the study population, may contribute to better adherence to the intervention.

**7. ETHICAL ASPECTS**

The study will follow the principles set out in the Declaration of Helsinki and the guidelines of Good Clinical Practice of the International Conference on Harmonization (ICH GCP). It also fulfills the requirements established in the legislative framework in Spain for the field of biomedical research, the protection of personal data and bioethics, as well as the published guidelines of good clinical practice.

The protocol has been evaluated by the Ethics Committee of IDIAP - Jordi Gol, incorporating its recommendations and suggestions.

During recruitment, all individuals will receive information about the study objectives and the activities related to their participation, and will sign an informed consent prior to their inclusion (Annex 1).

**8. AVAILABLE INSTRUMENTATION AND FACILITIES**

The research team has the institutional support of the ICS Directorate of Primary Care for Tarragona Province. During the project period, researchers will have the use of facilities belonging to ICS primary care centers in the cities of Tarragona and Reus and will have the technical resources at their disposal need for the study, including the blog and follow-up telephone calls. All the centers participating in the study function as primary care teams, with a common electronic medical registry system (e-CAP) managed by ICS. All PCCs have the physical space necessary for the laboratory tests to be carried out, in a confidential environment for the patients.

This project will also count on the collaboration of staff assigned to the Research Support Unit of the ICS Primary Care Area of ​​Tarragona-Reus, which has the computing resources required to manage the data generated by the study. The economic and administrative management of the project will rely on the infrastructure of IDIAP - Jordi Gol, which includes the Study Group on Pathology of the Respiratory System. In addition, the Primary Care Area of ​​Tarragona-Reus, through its research coordinator, is a member of the board of the Pere Virgili University Institute Board (Rovira i Virgili University, Tarragona), which provides access to the methodological and technical resources of these institutions.

**9. FUNDING SOURCES**

This project fits within the Strategic Plan for Research and Innovation in Health (Catalan acronym, PERIS) for 2017-2019, with funding granted by the Department of Health of the Government of Catalonia.

**10. REFERENCES**

1. Estruch R et al. Primary prevention of cardiovascular disease with a Mediterranean diet. N Engl J Med. 2013;368:1279–90.
2. Vieira AR et al. Fruits, vegetables and lung cancer risk: a systematic review and meta-analysis. Ann Oncol. 2016;27:81–96.
3. Hirayama F et al. Dietary factors for chronic obstructive pulmonary disease: epidemiological evidence. Expert Rev Respir Med. 2008;2:645–53.
4. Romieu I. Nutrition and lung health. Int J Tuberc Lung Dis. 2005;9:362–74.
5. Kohansal R et al. The natural history of chronic airflow obstruction revisited: an analysis of the Framingham offspring cohort. Am J Respir Crit Care Med. 2009;180:3-10.
6. Bentley AR et al. Dietary antioxidants and forced expiratory volume in 1s decline: the Health, Aging and Body Composition study. Eur Respir J. 2012;39:979-84.
7. Berthon B et al. Nutrition and Respiratory Health–Feature Review. Nutrients. 2015;7:1618–43.
8. Ng TP et al. Dietary and supplemental antioxidant and anti-inflammatory nutrient intakes and pulmonary function. Public Health Nutr. 2014;17:2081–6.
9. Garcia-Larsen V et al. Ventilatory function in young adults and dietary antioxidant intake. Nutrients.2015;7:2879–96.
10. Shahar E et al. Dietary n-3 polyunsaturated acids and smoking-related chronic obstructive pulmonary disease. Am J Epidemiol. 2008;168:796–801.
11. Jiang R et al. Cured meat consumption, lung function, and chronic obstructive pulmonary disease among United States adults. Am J Respir Crit Care Med. 2007;175:798–804.
12. Okubo H et al. Processed meat consumption and lung function: modification by antioxidants and smoking. Eur Respir J. 2014;43:972–82.
13. Sisson JH. Alcohol and airways function in health and disease. Alcohol. 2007;41:293–307.
14. Siu ST et al. Alcohol and lung airways function. Perm J. 2010;14:11–8.
15. Hu FB. Dietary pattern analysis: a new direction in nutritional epidemiology. Curr Opin Lipidol. 2002;13:3–9.
16. Varraso R et al. Prospective study of dietary patterns and chronic obstructive pulmonary disease among US women. Am J Clin Nutr. 2007;86:488–95.
17. Varraso R et al. Prospective study of dietary patterns and chronic obstructive pulmonary disease among US men. Thorax. 2007;62:786–91.
18. Shaheen SO et al. The relationship of dietary patterns with adult lung function and COPD. Eur Respir J. 2010;36:277–84.
19. Martin F et al. Multicentric randomized clinical trial to evaluate the long-term effectiveness of a motivational intervention against smoking, based on the information obtained from spirometry in primary care: the RESET study protocol. BMC Fam Pract. 2016;4;17:15.
20. Sorli M et al. Dietary patterns are associated with lung function among Spanish smokers without respiratory disease. BMC Pulm Med. 2016 (in press).
21. Sorlí M et al. Adiposity markers and lung function in smokers: a cross-sectional study in a Mediterranean population. BMC Pulm Med 2016, in press.
22. Davis C et al. Definition of the Mediterranean Diet: A Literature Review. Nutrients. 2015;7:9139-53.
23. Campbell M et al. Sample size calculations for cluster randomised trials. Changing Professional Practice in Europe Group (EU BIOMED II Concerted Action). J Health Serv Res Policy. 2000;5:12-6.1078758.
24. Vallbona Cl et al. “Guia de prescripció d’exercici físic per a la salut” (PEFS), Dirección General de Salud Pública (Departamento de Salud) y Secretaria General del Deporte (Departamento de la Vicepresidencia), Generalitat de Catalonia. PDF available at: [http://www20.gencat.cat](http://www20.gencat.cat/docs/canalsalut/Home%20Canal%20Salut/Professionals/Temes_de_salut/Activitat_fisica/documents/Guiadeprescripcioversioextensa.pdf). [Accessed 01/06/2017]
25. Trinidad I et al. Validation of a short questionnaire on frequency of dietary intake: reproducibility and validity. Nutr Hosp. 2008;23:242-52.
26. Schröder H et al.A short screener is valid for assessing Mediterranean diet adherence among older Spanish men and women. J Nutr. 2011;14:1140-5.
27. Miller MR et al. Standardisation of spirometry. Eur Respir J. 2005;26:319–38.
28. Look AHEAD Research Group, Wing RR, Bolin P, Brancati FL, Bray GA, Clark JM, et al. Cardiovascular effects of intesive lifestyle intervention in type 2 siabetes. N Engl J Med. 2013; 369: 145-54.

**11. ANNEXES AND FIGURES**

**Annex 1. Informed Consent**.

**INFORMATION SHEET FOR PARTICIPANTS**

**(TRANSLATION)**

**MEDISTAR clinical trial (Mediterranean Diet and Smoking in Tarragona and Reus). Effect of the Mediterranean Diet on lung function in smokers without previous respiratory pathology**.

**Principal investigator**

Francisco Martín Luján.

Directorate of Tarragona-Reus Primary Care Area, Catalan Institute of Health.

**Objective**

To evaluate the effect of Mediterranean Diet on the deterioration of lung function in smokers without respiratory disease.

**Methodology and study development**

A total of 20 primary care centers in Tarragona are participating in this study. The intention is to include a total of 566 active smoking individuals who meet the inclusion criteria and agree to participate voluntarily. Once included in the study, participants will be divided into two groups (control group and intervention group). One group will continue to follow their usual diet; the intervention group will be asked to follow the Mediterranean Diet.

**Intervention**

All people will be interviewed at the beginning of the study and on several follow-up visits during two years. Researchers will collect information about their dietary habits and provide nutritional information. We will evaluate and compare how lung function changes in both groups. At the end of the study, if the intervention is effective, control group members will be invited to participate in the same intervention program.

**Analysis of biological markers**

In order to compare dietary adherence, in addition to the questionnaires, several “biomarkers” will be tested as a direct measure of food consumption. Therefore, this consent form includes the authorization to obtain blood and urine samples, which will be used to analyze these biological markers (such as oleic acid, resveratrol, and hydroxytyrosol), which indicate a response to a nutritional intervention. We will also analyze some markers of inflammation and oxidation that may be related to the diet.

The use and storage of the samples for the study has been approved by the Committee for Ethics and Clinical Research of the Institute for Research in Primary Care (IDIAP) Jordi Gol.

**Can I see the results of the biological markers tests?**

You have the right to the data obtained from your sample if you request it. In any case, if a test result is relevant to your health, you will be informed through your primary care physician.

**Adverse Events**

No adverse effects are anticipated. Any health problem that occurs during the study period will be attended according to the usual clinical practice in your Primary Care Center. Study participation will be suspended if any other health priority affects your involvement.

**Voluntary participation**

Your participation in this study is completely voluntary and you can withdraw from it at any time, without having to give any explanation and without this affecting your medical care or your relationship with your usual health care team.

**Confidentiality**

All your personal and biological data will be considered confidential and treated with the level of protection (RD 1720/2007) required for this type of data according to the Organic Law of Protection of Personal Data (15 /1999).

The samples obtained and their data will be protected with a code. The key to the codes will be in sole custody of the principal investigator of the study, who guarantees absolute confidentiality.

The results of the study may be communicated to the scientific community in the context of seminars or conferences, or published in scientific articles. However, the identity of the participants will not be revealed in any publication or report related to this study.

**Additional Information**

If you have questions about any aspect of the study or you would like to discuss any additional questions, you can contact the dietitian, Patricia Salamanca, at 977 778 518 or by sending an email to [medistar.tgn.ics@gentcat.cat](mailto:medistar.tgn.ics@gentcat.cat). Having read this information and clarified possible doubts, if you decide to participate, you must sign the following informed consent.

**INFORMED CONSENT SHEET**

**MEDISTAR (Mediterranean Diet and Smoking in Tarragona and Reus)**

**Effect of the Mediterranean Diet on lung function in smokers without previous respiratory pathology.**

**Principal investigator**

Francisco Martín Luján.

Directorate of Tarragona-Reus Primary Care Area, Catalan Institute of Health.

Mr. / Mrs. (Name and surname of the **participant**):

____________________________________________________________________

I declare that:

• I have been informed by (name and surname of the **researcher**):

_____________________________________________________________________

I confirm the following:

**• I have read the information sheet that has been given to me.**

**• I have been able to ask questions about the study.**

**• My questions have been answered.**

• I have been **sufficiently informed,** I understand that my participation is voluntary, and I understand that I can withdraw from the study at any time, without having to give any explanation and without affecting my medical care.

With respect to analysis of biochemical markers:

**• I agree to participate voluntarily in the analysis of biological markers by the MEDIS·TAR study.**

Therefore, I freely agree and sign my consent to participate in the MEDIS·TAR study.

Signature (Participant):

In (location) __________________, as of date: ____ / ____ / ______

**Figure 1. Project flowchart: participant selection, randomization and follow-up.**

**Visit 5 (24 months)**

Data collection

**INTERVENTION Group**

Mediterranean diet

Anti smoking advice

(10 centers)

Access to a nutritional blog, *ad hoc*

**Exclusion of patients who do not wish to participate or do not meet the inclusion criteria.**

**Visit 2 (6 months)**

Telephone call to reinforce message

**Visit 4 (18 months)**

Telephone call to reinforce message

**Visit 5 (24 months)**

Data collection

**Visit 3 (12 months)**

Data collection

Nutrition intervention

**Visit 1 (baseline)**

Data collection

Nutrition intervention

**MEDISTaR Study**

Smokers from 25 to 75 years old / 20 PCCs in Tarragona Province

**Visit 3 (12 months)**

Data collection

**Visit 1 (baseline)**

Data collection

**CONTROL Group**

Usual diet

Anti smoking advice

(10 centers)

**ANALYSIS OF DATA**

REPORTING

**Visit 0 (pre-selection and inclusion of patients)**

Obtain signed informed consent for the study.

Randomize participants by clusters (PCCs)
